# Genetic mapping of EgfrL.3.1 in Drosophila melanogaster

**DOI:** 10.17912/micropub.biology.000098

**Published:** 2019-04-26

**Authors:** Joyce Stamm, Gnanda S Joshi, MA Anderson, Katie Bussing, Colton Houchin, Amber C Elinsky, Jacob T Flyte, Nadine Husseini, Dominika Jarosz, Chelsea L Johnson, Abby F Johnson, Christina E Jones, Taj P Kooner, Daniel Myhre, Thomas N Rafaill, Sarah Sayed, Kirby W Swan, Jonathan Toma, Jacob D Kagey

**Affiliations:** 1 Department of Biology, University of Evansville; 2 Biology Department, University of Detroit Mercy

**Figure 1.**
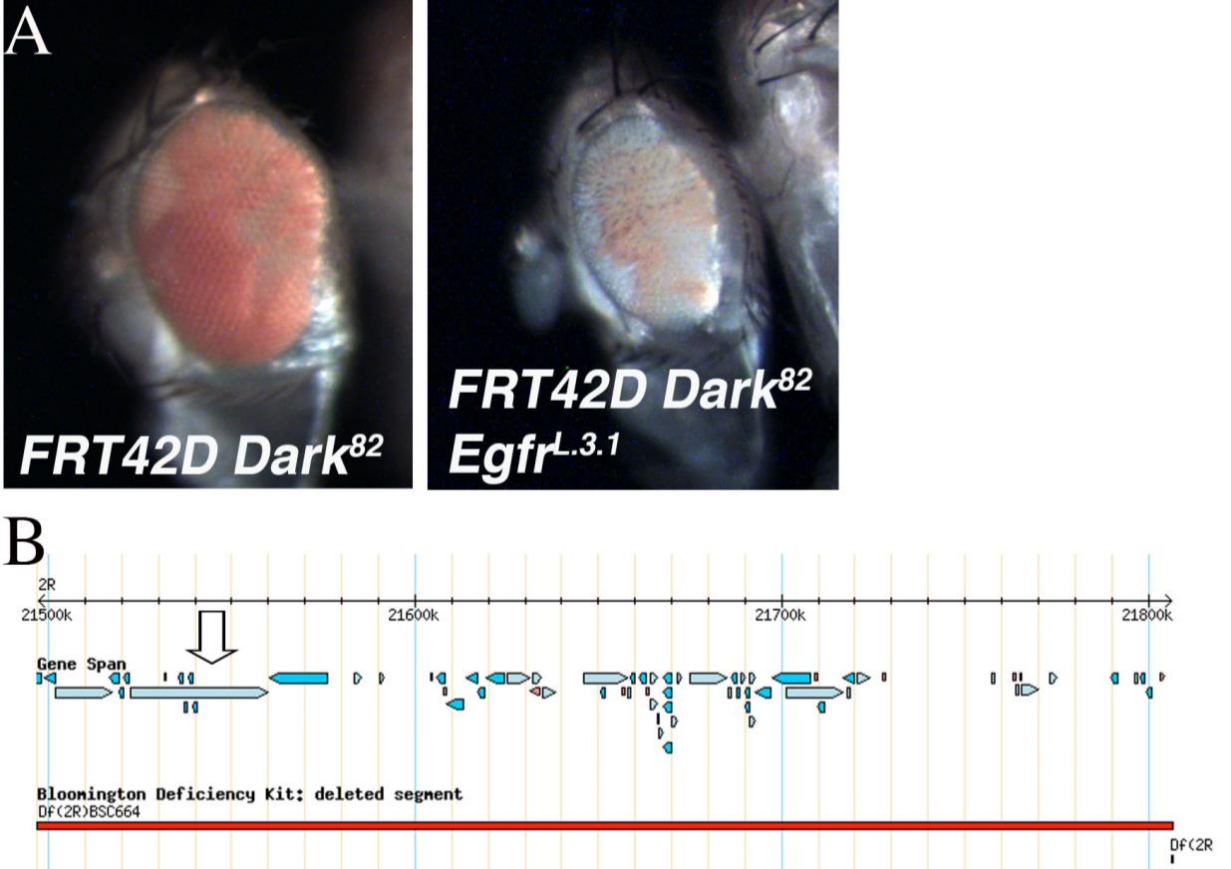
A. Mosaic (*FRT42D Dark^82^*), and *Dark^82^* *Egfr^L.3.1^* (*FRT42D* *Dark^82^Egfr^L.3.1^*) eyes. In both eyes the homozygous mutant tissue is pigmented (*w^+mC^*). B. Region of chromosome 2R that failed to complement L.3.1 by deficiency mapping (2R:21,497,290..21,806,350). Arrow denotes location of *Egfr* gene. B is adapted from flybase.org (Gramates et al., 2017).

## Description

An EMS screen was conducted utilizing the Flp/FRT system to identify mutations that caused an array of phenotypic alterations in the size of the eye including the ratio of mutant to wild type tissue (red over white) or the developmental patterning of the mosaic eye. This screen was done in the genetic background of blocked apoptosis in the homozygous mutant cells to identify conditional regulators of cell growth and eye development (Kagey *et al.,* 2012). The block in apoptosis in the mosaic mutant tissue was achieved by using a *FRT42D Dark^82 ^*chromosome as a starting point for the EMS mutagenesis (Akdemir *et al.,* 2006). The *Dark^82^* allele was generated by an imprecise excision of the P{lacW}Ark^CD4^, this allele retains the *w^+mC^* (Akdemir *et al.,* 2006) One of the mutants identified was *L.3.1* which generated a small rough eye mosaic phenotype, with a smaller percentage of pigmented tissue than the *FRT42D, Dark^82 ^*control (Figure 1A). The *Dark^82 ^*mosaic eye is approximately 60% pigmented tissue, while the *Dark^82 ^L.3.1* mosaic eye was smaller overall and approximately 50% mutant tissue (*w^+mC^*). The ‘rough eye’ phenotype indicates a disruption in the ommatidial organization. In both images the pigmented (*w^+mC^*) tissue is homozygous mutant and the unpigmented tissue is homozygous wild type.

The genetic mapping of the location of mutant *L.3.1* was done by two independent groups of undergraduate researchers at the University of Detroit Mercy and University of Evansville in undergraduate genetics laboratory courses as part of the Fly-CURE consortium (Bieser *et al.,* 2018). Complementation mapping was conducted independently and the results confirmed between groups. Virgin females from the *FRT42D L.3.1 Dark^82^/CyO* stock were mated in series to male flies from the 87 deficiency stocks that comprise the Bloomington Stock Center 2R Deficiency Kit (only stocks distal to the FRT42D site were used for mapping) (Cook *et al.,* 2012). Mutant *L.3.1* failed to complement Deficiency stock Df(2R)BSC664 (2R:21,341,647..21,872,028), while complementing the flanking overlapping deficiencies Df(2R)BSC821 and Df(2R)BSC597. This left a region of failure to complement of 2R:21,497,290..21,806,350, which is pictured above in Figure 1 B. Lethal alleles of candidate genes within this region were mated independently to *L.3.1* to test for complementation. *L.3.1* failed to complement an apparent loss of function allele *Egfr^k05115^ (*Dworkin *et. al. 2006),* indicating that *L.3.1* is likely a novel *Egfr* allele, *Egfr^L.3.1^.*

## Reagents

*FRT42D Dark^82^/CyO* (Akdemir et al., 2006)

*FRT42D Dark^82^ Egfr^L.3.1^/CyO* (this manuscript)

*Ey>Flp; FRT42D* (BDSC 5616)

*y^1^ w^67c23^*; P{*w^+mC^*=lacW}*Egfr^k05115^*/CyO (BDSC 10385)

Bloomington Drosophila Stock Center 2R Deficiency Kit (Cook *et al.,* 2012):

*w^1118^;* Df(2R)BSC664/SM6a

*w^1118^*; Df(2R)BSC821, P+PBac{ *w^+mC^* =XP3.RB5}BSC821/SM6a

*w^111^];* Df(2R)BSC597/SM6a
